# Is Thermal Imaging a Helpful Tool in Diagnosis of Asymptomatic Odontogenic Infection Foci—A Pilot Study

**DOI:** 10.3390/ijerph192316325

**Published:** 2022-12-06

**Authors:** Daria Wziątek-Kuczmik, Iwona Niedzielska, Aleksandra Mrowiec, Karolina Bałamut, Maciej Handzel, Agnieszka Szurko

**Affiliations:** 1 Department of Cranio-Maxillofacial Surgery, Faculty of Medical Sciences, Medical University of Silesia, 40-027 Katowice, Poland; 2 Faculty of Science and Technology, University of Silesia, 40-752 Katowice, Poland

**Keywords:** thermography, odontogenic infection foci, concomitant disease

## Abstract

Since the identification of periapical lesions typically requires invasive testing that may adversely affect individuals suffering from concomitant disease, the diagnosis of apical periodontitis remains a challenge. This study aimed to determine the effectiveness of infrared thermal imaging for the detection of asymptomatic odontogenic inflammatory response in patients with a high risk of systemic infections. The examinations were performed using the FLIR T1020 thermal camera. The acquired images were analyzed with a ThermaCAM TM Researcher Pro 2.8 SR-3. Statistical analyses were conducted using the Statistica 10 software. The Mann–Whitney U test was used for data that were not normally distributed or did not meet the assumption of homogeneity of variance, while normally distributed data were analyzed with the t-test. The mean temperature difference between the periapical regions of the suspect and contralateral teeth was found to be greatest at 30 s of mouth opening. This is a preliminary study conducted to evaluate the potential of infrared thermal imaging as a diagnostic tool for the identification and elimination of odontogenic infection foci. Thermography seems to facilitate the quantitative assessment of inflammation by displaying temperature differences between the affected and unaffected regions.

## 1. Introduction

Thermography is a modern imaging method in which the infrared radiation emitted by an object allows the mapping and analysis of the exact temperature distribution on the surface of the object [1,2]. It involves non-contact temperature measurement using thermal imaging cameras and subsequent image analysis. Thermal images depict changes in body temperature that may correlate with changes with local blood supply and tissue metabolism. The intensity of the infrared radiation depends on the temperature of an object. The obtained thermograms are presented in a pseudo-color projection, which facilitates the image quality analysis [3,4,5].

Thermovision in dentistry is already widely used and, thus, for example, it becomes a helpful tool in the analysis of periodontium or temporomandibular joints (TMD) [6,7,8]. Thermovision is also useful in assessing the condition and distinguishing living from dead teeth [9].

In maxillofacial surgery, it is used to diagnose odontogenic foci of infection, cancers of the oral cavity and diseases of the maxillary sinuses and salivary glands [10], or to assess postoperative inflammation after tooth extraction [11]. Thermography has also been used to assess pain in patients after tooth extraction [12] or to characterize chronic orofacial pain. IRT is also used to assess the progress of socket healing after therapies such as photobiomodulation therapy (PBMT) [11] or photodynamic therapy (PDT) [13]. Thermography is also used to assess the condition of thermally damaged dental pulp [14] after the use of the Electro Thermal Debonding (ETD) method.

The introduction of infrared thermal imaging to medicine required experimental confirmation of the correlation between the variability of the blood supply to the area of interest and the resulting temperature changes. Thermal maps specific to a given pathology were then developed. Thermal asymmetry in a given area may be sufficient to diagnose dysfunction.

The advantage of this type of imaging is the fact that it is a non-invasive method, which is why it has been successfully used in medicine for several dozen years. Its benefits are also noticed and used by specialists in biomedical engineering, medical physics and physiotherapy. It is mainly a supplementary method to diagnostic tests, but thanks to the availability of more and more accurate devices that allow for the analysis of the temperature distributions of the body surface and the development of new methods of analyzing the obtained thermal images, it can be regarded as a developing method with great potential [2,3,4,5,15,16,17,18,19,20,21].

The aim of this study was to determine the effectiveness of infrared thermal imaging in detecting asymptomatic, odontogenic inflammatory responses in patients at high risk of systemic infections.

## 2. Materials and Methods

Pilot studies aimed at developing a proprietary, non-invasive method of identifying asymptomatic foci of odontogenic infection, in people at high risk of systemic infection, i.e., patients with hematopoietic disorders, cardiovascular or urinary tract diseases, eye diseases, maxillary sinus diseases and other diseases, along with those waiting for a kidney transplant or an autologous/allogeneic bone marrow transplant, were performed at the Cranio-Maxillofacial Surgery Clinic of the Medical University of Silesia from September to December 2021. The study was approved by the Ethics Committee of the Medical University of Silesia (approval number PCN/CBN/0052/KB1/67/I/22).

During study recruitment, all patients underwent diagnostic tests or disease treatment; they did not report dental abnormalities but were referred for dental assessment to eliminate any pathology of odontogenic origin. Including a patient in the study group was based on a strict algorithm. Physical examination was of key importance; the medical records of each patient, as well as the results of laboratory tests and diagnostic imaging, were carefully analyzed. All allergies, pharmacotherapies and the use of caffeine, alcohol and other stimulants were recorded. Temperature, blood pressure and heart rate readings were taken; body height and weight were measured and BMI was calculated.

In order to identify a potential odontogenic infection focus, each patient underwent a thorough extra- and intraoral examination. The latter comprised careful assessment of the periodontal tissues and oral mucosa. All suspect teeth were inspected using a number of diagnostic tests.

The radiographic examination included panoramic and intraoral radiographs. If necessary, the patient was referred for X-ray of paranasal sinuses or cone beam CT (CBCT).

The inclusion criteria included an asymptomatic pathology associated with odontogenic tissues diagnosed on the clinical and/or radiographic examination, the presence of the healthy contralateral tooth. The study group comprised adult male and female patients with a concomitant disease.

Since it was a pilot study, the initial thermographic analysis concerned asymptomatic odontogenic infection foci, such as:

1. Teeth or teeth roots with pulp necrosis or gangrenous teeth—both with or without periapical lesions (compared to healthy contralateral teeth);
2. Endodontically treated teeth with or without periapical lesions (compared to healthy contralateral teeth);
3. Teeth with periodontal disease (compared to healthy contralateral teeth).

Each patient underwent infrared thermography after appropriate pre-test preparation in an imaging room conforming to attributes required for thermal image acquisition. Before imaging, the participants rested for approximately 30 min to achieve thermal acclimatization. They were instructed not to use stimulants, engage in exercise or strenuous physical activity, use the sauna or sunbathe on the day of their appointment. Medications prescribed for concomitant disease were allowed.

Eight cases (each with a concomitant disease) diagnosed with potential odontogenic infection foci in the maxilla or mandible were recruited. As already mentioned, it was required that each tooth with pathology should have a healthy contralateral tooth, i.e., without advanced carious or periapical lesions on X-rays. 

A total of 21 teeth were analyzed. Among them were 16 endodontically treated teeth, including 5 teeth with periapical lesions and 1 with root end cyst. There were 2 leftover roots with periapical lesions and 2 dead teeth with periapical lesions, while 1 dental implant was also analyzed. 

The control group consisted of 12 healthy (i.e., without concomitant disease) volunteers who underwent thermal imaging of the periapical region of the suspected and healthy contralateral teeth. 

The examinations were performed using the FLIR T1020 thermal camera in a temperature-controlled room (23.0 ± 1 (°C)) with a humidity of 50%. A skin emissivity value of 0.98 was used. The camera was perpendicular to the skin surface; the distance between the camera and the body was 0.4 ± 0.05 m, all according to the standards of infrared medical diagnosis. Prior to imaging, the participants stayed in a resting position to achieve thermal acclimatization (the minimum acclimatization time was 20 min) [8]. 

The acquired images were analyzed with a ThermaCAM TM Researcher Pro 2.8 SR-3. Statistical analyses were conducted using the Statistica 10 software. The Mann–Whitney U test was used for data that were not normally distributed or did not meet the assumption of homogeneity of variance, while normally distributed data were analyzed with the t-test.

Immediately before the examination, patients rinsed their mouths with room temperature water for 1 min. Thereafter, the patient’s head was fixed in a position with the mouth open and lip retractors in place. Thermal images of the suspect teeth in the anterior segment of the maxilla/mandible were compared with those of the contralateral teeth. Molars were excluded from measurements as their anatomical location makes image acquisition difficult. 

The camera was positioned perpendicular to the subject in the sagittal plane; the tooth apex was at the center of the investigated area. The mouth was open for approximately 120 s, with thermographic images of cooling intraoral tissues captured at 30 s intervals. Images of the healthy contralateral teeth were acquired in the same way. The mean temperature of 1 cm^2^ at the selected apex region of the right and left maxillary and mandibular arches was noted.

## 3. Results

**Figure 1:**
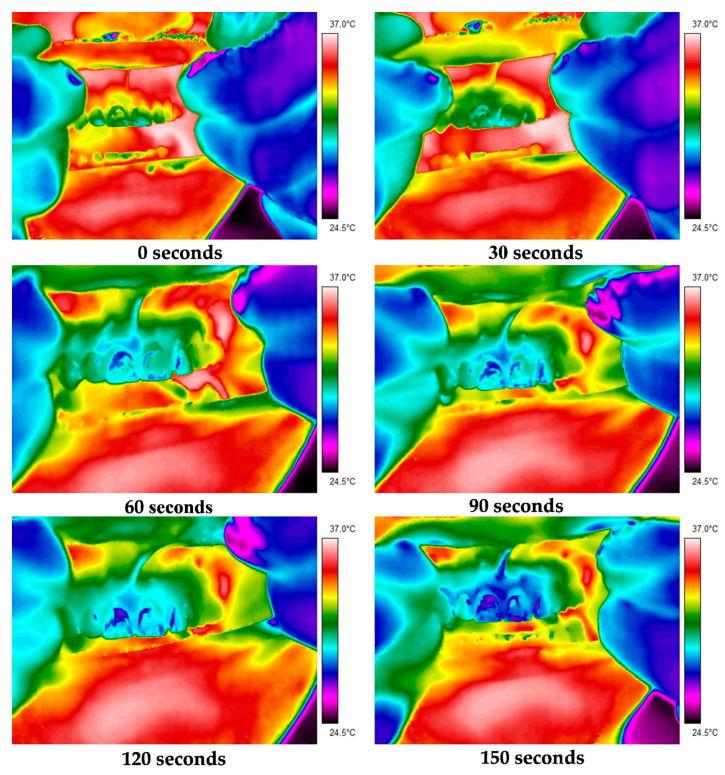
Thermal images of patient I: teeth 21 and 22 after endodontic treatment without periapical lesions; contralateral healthy teeth 11 and 12.

**Figure 2:**
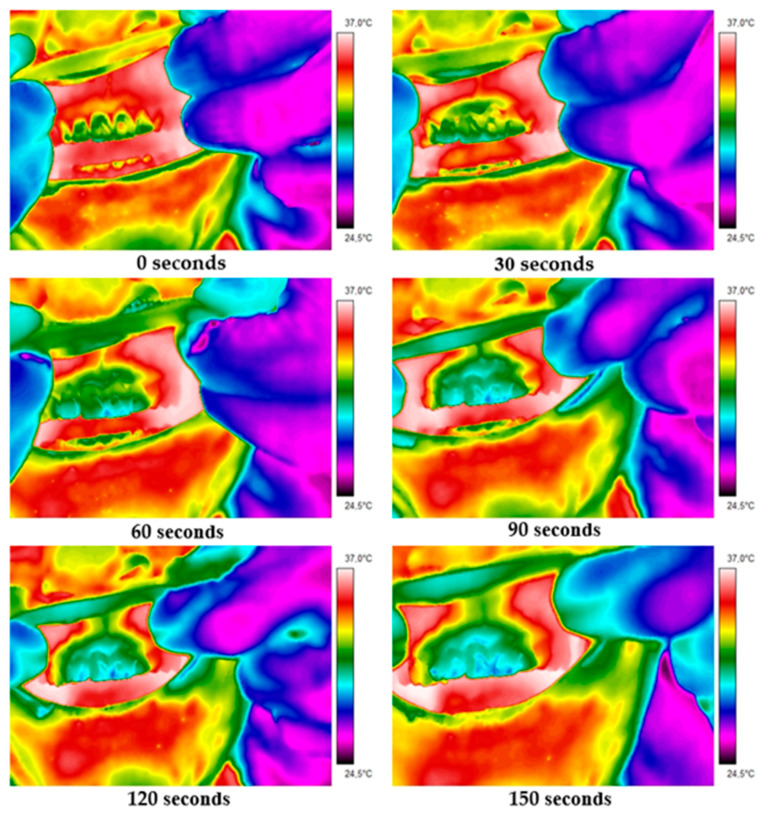
Thermal images of patient II obtained just after opening the mouth and at 30, 60, 90, 120 and 150 s after. There are two teeth imaged—tooth 22 after resection and contralateral healthy tooth 12.

Detailed thermal parameters of the suspect and contralateral healthy teeth (Table 1) were read from the obtained thermograms. Figure 1 and Figure 2 depict thermograms acquired in two representative patients. Thermal imaging was performed for 120 s, with images of cooling intraoral tissues captured at 30 s intervals. Images of the healthy contralateral teeth were acquired in the same way.

**Figure 3:**
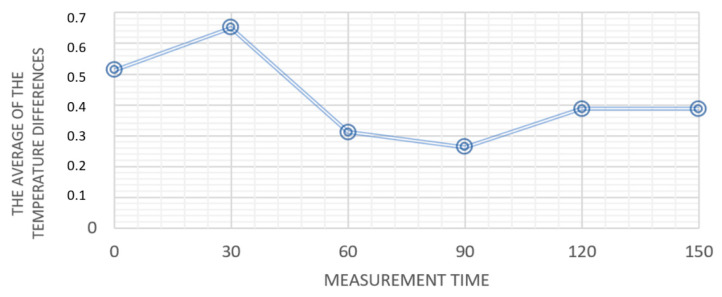
Mean temperature differences in the periapical regions of the suspect and contralateral teeth obtained for all patients, with the time of measurement during the patient keeping mouth open.

The mean differences between the average temperatures derived from the thermal images in the periapical regions of the suspect and contralateral teeth are presented in Figure 3.

Since the aim of this pilot study was to develop a screening thermal imaging test for intraoral diagnosis, we focused on the differences between the suspect teeth and their healthy counterparts. The obtained thermal images indicated temperatures in the periapical regions of the suspect teeth (with periapical lesions, cysts or after adequate endodontic treatment) differed from those in the corresponding regions of the contralateral healthy teeth. It seems that the absolute value of the difference between the mean periapical temperatures of the suspect and contralateral teeth might serve as a thermal parameter to help identify intraoral pathologies. The mean temperature difference between the compared periapical regions was the greatest at 30 s of mouth opening. 

**Figure 4:**
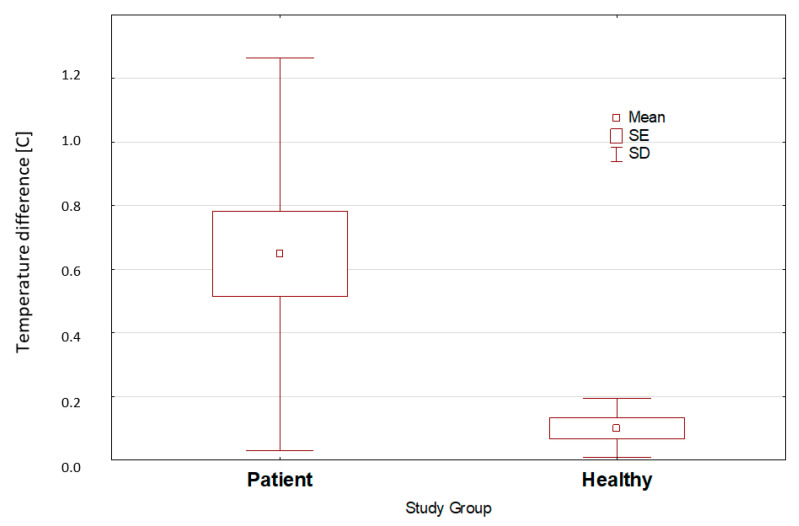
Box-and-whisker plot—absolute values of the mean periapical temperature differences in the suspect and contralateral teeth of the study and control group at 30 s of mouth opening.

The Mann–Whitney U test revealed the differences between the absolute values of the mean periapical temperatures of the suspect and contralateral teeth were statistically significant (Figure 4).

The absolute values of the mean periapical temperature of the suspect and contralateral teeth differed significantly between the study and control groups (*p* < 0.01). The respective absolute values were 0.65 and 0.1 for the patients and control groups.

## 4. Discussion

Our study showed that infrared thermography can serve as a complementary examination to routinely used dental imaging techniques or even as an important screening test. Thermography is a non-invasive, non-contact approach, is safe for the patient and the examiner, and can be used regardless of sex and age [22]. It seems useful for the diagnosis of asymptomatic odontogenic infection foci in patients at high risk of systemic infections. So far, such foci have been identified using highly invasive tools, such as a histamine or penicillin challenge test or electric tooth vitality test, which pose a risk to patients with systemic disease [23]. Therefore, there is still a search for effective and safer methods of diagnosing subclinical infections, assessing their dynamics and preventing systemic spread. Thermal imaging may facilitate the identification of silent infection foci, and thus contribute to the timely diagnosis and treatment of concomitant diseases. It may also help raise awareness regarding the impact of oral health on systemic health among patients and medical doctors, some of whom still doubt whether dental disease could be associated with systemic disease. 

The primary foci of odontogenic infections are teeth with necrotic pulp, pulpless teeth and endodontically treated teeth with inadequate root filling. Other causes include apical periodontitis, dental cysts, apical surgery failure, osteitis, leftover tooth roots/fragments or impacted teeth. Periodontitis may also contribute to the development of odontogenic inflammatory response [23]. All these disorders tend to change the temperature of the affected tissues; hence, the turn towards diagnostic thermography.

Inflammation results from the response of the immune system to disorders of hemostasis. At the tissue level, inflammation is characterized by redness, swelling, temperature elevation, pain and loss of function caused by local vascular and inflammatory responses to infection. On the other hand, the stages of apical periodontitis (from the acute phase to complete necrosis) can be asymptomatic. According to the American Association of Endodontists, the initial stages of pulpal necrosis do not produce clinical signs/symptoms or radiographic signs. For this reason, and also due to multifactorial etiology, the diagnosis of periapical inflammatory lesions remains a challenge—even for experienced professionals. 

No correlation was revealed between the clinical and radiographic findings characteristic of a primary inflammatory infiltrate. It can therefore be speculated that this type of infiltration was not correlated with the symptomatic status of the patient, unlike the secondary infiltration. The latter might be associated with pain and edema caused by exacerbation of a chronic inflammatory condition. The clinical symptoms of periapical lesions develop over time with disease progression and depend on irritants accumulated in periapical tissues, including bacterial fragments, toxins, products of pulp tissue degradation, microorganisms and external factors such as systemic conditions [24,25]. Local changes in tissue temperature strongly suggest changes in tissue metabolism and blood supply typical of a given pathology. These processes seem to be dynamic; therefore, absolute differences in thermal parameters should be noted, at least during the initial diagnostic assessment.

The ability of thermal imaging to identify temperature changes associated with various pathological conditions results from amazing technological advancement in this field. A new generation of infrared cameras can detect changes of 0.02 °C that may reflect pathophysiological pathways underlying specific inflammatory conditions. Numerous studies [10,22,25,26,27,28,29,30,31] have shown that thermal images of body surfaces represent the variability of blood flow and metabolism in an area of interest and can be used to identify a wide range of pathologies. 

Numerous authors have suggested that thermal imaging might complement the routinely used dental imaging techniques. Paredes et al. [32] conducted a study to develop a thermography protocol for assessment of dental pulp and periapical tissues vascularization. The ultimate aim was to develop a non-invasive pulp vitality diagnostic test. The thermal and electric tests used so far can yield false-positive results [32]. Mendes et al. used infrared thermography to quantify and compare the temperature of vital and non-vital anterior teeth and concluded that these temperatures differed significantly [9]. It should be noted though that the temperature of a diseased tooth (or its periapical region—images of teeth are not acquired) will not always be higher than that of the healthy contralateral tooth. Therefore, we suggest absolute values of the mean temperature differences should be analyzed for the suspect and contralateral healthy teeth. We also analyzed the effect of time lapse between mouth opening (start of the tissue cooling process) and image acquisition. Our findings showed temperature differences between the periapical regions of the suspect and healthy teeth were the greatest at 30 s of mouth opening. However, it should be noted that the biggest thermal differentiation effect is not permanent, so the time of imaging after opening the mouth should be measured and the environment condition in the measurement room should be stable. This might become a new standard in thermal imaging and prove valuable for oral health professionals who use infrared thermal cameras for intraoral diagnosis.

## 5. Conclusions

This paper presented a pilot study aimed at using an infrared thermal imaging camera as a simple and non-invasive diagnostic tool to identify odontogenic infection foci. Absolute values of mean temperature differences between the periapical regions of suspect and contralateral healthy teeth were analyzed. The mean temperature difference between the periapical regions of the suspect and contralateral teeth was found to be the greatest at 30 s of mouth opening. The difference between temperature values obtained for suspect and contralateral teeth in the study group and similar measurements performed for the control group was statistically significant. 

Thermography seems to facilitate quantitative assessment of inflammation by displaying temperature differences between the affected and unaffected regions. It helps identify subclinical lesions and can therefore prove useful in clinical research studies. 

This was a preliminary study conducted to evaluate the potential of infrared thermal imaging as a diagnostic tool for identification of odontogenic infection foci.

## Figures and Tables

**Table 1:** Periapical regions of the suspect and contralateral healthy teeth—thermograms captured at 30 s intervals in five patients of the study group.

	Periapical Region, Tooth Number	Mean Temp (°C) at 0 s	Mean Temp (°C) at 30 s	Mean Temp (°C) at 60 s	Mean Temp (°C) at 90 s	Mean Temp (°C) at 120 s	Mean Temp (°C) at 150 s
Patient I	21	34.6	33.5	32.3	31.7	32.1	31.2
	11	33.9	32.9	32.1	31.7	31.9	31.1
	Temp difference (°C)	0.7	0.6	0.2	0	0.2	0.1
	22	34.9	34.5	32.6	32.1	32.4	31.4
	12	34.5	33.3	32.6	31.9	32.4	31.7
	Temp difference (°C)	0.4	1.2	0	0.2	0	0.3
Patient II	22	35	34.5	33.6	33.4	33.4	33.4
	12	34.6	34	33.1	32.5	32.4	32.4
	Temp difference (°C)	0.4	0.5	0.5	0.9	1	1
Patient III	14	35.8	34.6	35.2	35.1	35.2	35.4
	24	35.6	35.4	35	35	35	35.2
	Temp difference (°C)	0.2	0.8	0.2	0.1	0.2	0.2
Patient IV	12	35.3	32.6	32	32.2	32.2	33.1
	22	34.6	33.3	32.5	32.4	31.9	33.1
	Temp difference (°C)	0.7	0.7	0.5	0.2	0.3	0
	11	35.8	33.4	33.3	32.8	32.7	33.7
	21	34.3	33.3	32.6	32.4	31.8	33.1
	Temp difference (°C)	1.5	0.1	0.7	0.4	0.9	0.6
Patient V	21	35.3	31.9	32	32.5	32	32.6
	11	35.3	32.4	32.1	32.5	32.3	32.7
	Temp difference (°C)	0	0.5	0.1	0	0.3	0.1
	22	35.6	33.5	33	33.1	33.2	33.1
	12	35.8	34.3	33.3	33.4	33.4	33.9
	Temp difference (°C)	0.2	0.8	0.3	0.3	0.2	0.8
	Mean temp difference (°C)	0.51	0.65	0.31	0.26	0.39	0.39

Patient I—(P.A.) teeth 21, 22 after endodontic treatment; no periapical lesions. Patient II—(B.K.) tooth 22 after endodontic treatment and tooth apex resection. Patient III—(A.B.) teeth 14, 15 periodontal disease and periapical lesions, periodontitis. Patient IV—(P.C.) teeth 11, 12 after endodontic treatment; no periapical lesions. Patient V—(M.K.) teeth 21, 22 after endodontic treatment; no periapical lesions.

## Data Availability

Not applicable.
